# A double-blind, placebo-controlled, randomised withdrawal study of adjunctive brexpiprazole maintenance treatment for major depressive disorder

**DOI:** 10.1017/neu.2024.32

**Published:** 2024-10-17

**Authors:** Roger S. McIntyre, Kripa Sundararajan, Saloni Behl, Nanco Hefting, Na Jin, Claudette Brewer, Mary Hobart, Michael E. Thase

**Affiliations:** 1 Brain and Cognition Discovery Foundation and University of Toronto, Toronto, ON, Canada; 2 Otsuka Pharmaceutical Development & Commercialization Inc., Princeton, NJ, USA; 3 H. Lundbeck A/S, Valby, Denmark; 4 Perelman School of Medicine, University of Pennsylvania and the Philadelphia Veterans Affairs Medical Center, Philadelphia, PA, USA

**Keywords:** Antipsychotic, clinical trial, major depressive disorder, Phase 3, relapse, withdrawing treatment

## Abstract

**Objective:**

To compare time to relapse in patients with major depressive disorder (MDD) stabilised on antidepressant treatment (ADT) + brexpiprazole who were randomised to continued adjunctive brexpiprazole or brexpiprazole withdrawal (switch to placebo).

**Methods:**

This Phase 3, multicentre, double-blind, placebo-controlled, parallel-arm, randomised withdrawal study enrolled adults with MDD and inadequate response to 2–3 ADTs. All patients started on adjunctive brexpiprazole 2–3 mg/day (Phase A, 6–8 weeks). Patients whose symptoms stabilised (Phase B, 12 weeks) were randomised 1:1 to adjunctive brexpiprazole or adjunctive placebo (Phase C, 26 weeks). The primary endpoint was time to relapse in Phase C. Depression rating scale score changes were secondary endpoints.

**Results:**

1149 patients were enrolled and 489 patients were randomised (ADT + brexpiprazole *n* = 240; ADT + placebo *n* = 249). Median time to relapse was 63 days from randomisation in both treatment groups for patients who received ≥1 dose. Relapse criteria were met by 22.5% of patients (54/240) on ADT + brexpiprazole and 20.6% (51/248) on ADT + placebo (hazard ratio, 1.14; 95% confidence interval, 0.78–1.67; *p* = 0.51, log-rank test). Depression scale scores improved during Phases A–B and were maintained in Phase C. Mean weight increased by 2.2 kg in Phases A–B and stabilised in Phase C.

**Conclusion:**

Time to relapse was similar between continued adjunctive brexpiprazole and brexpiprazole withdrawal; in both groups, ∼80% of stabilised patients remained relapse free at their last visit. Adjunctive brexpiprazole therapy was generally well tolerated over up to 46 weeks, with minimal adverse effects following brexpiprazole withdrawal.

ClinicalTrials.gov identifier: NCT03538691. Funding: Otsuka, Lundbeck.

## Graphical abstract

**Figure:**
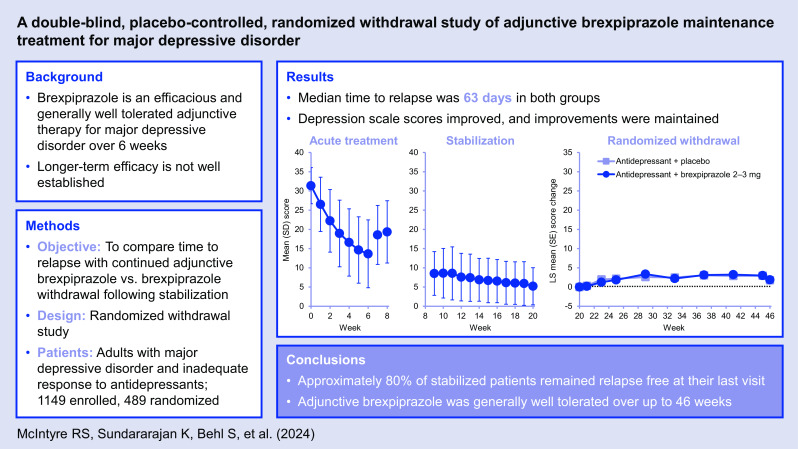

## Significant outcomes

- After 18–20 weeks of treatment, discontinuation of adjunctive brexpiprazole in stabilised patients with major depressive disorder was associated with a low risk of relapse and minimal adverse withdrawal effects.
- Over 26 weeks following stabilisation, time to relapse was similar with continued adjunctive brexpiprazole and brexpiprazole withdrawal; early improvement of depressive symptoms and functioning was maintained in the majority of stabilised patients.
- Adjunctive brexpiprazole was generally well tolerated over up to 46 weeks.

## Limitations

- Participants received 18–20 weeks of adjunctive brexpiprazole therapy before randomisation; risk of relapse may have been higher if a shorter duration of adjunctive therapy was provided prior to randomisation.
- There was no active comparator arm (such as another adjunctive antipsychotic) to validate the design and conduct of the trial.
- Relapse rates were low in the group randomised to brexpiprazole withdrawal, which may suggest that the study lacked sensitivity.

## Introduction

Remission, defined as no or minimal depressive symptoms plus improved functioning, is the key goal of acute treatment for major depressive disorder (MDD) (Bauer *et al*., 2013). However, relapse rates following remission are high (Sim *et al*., 2016), and long-term studies show that most patients in recovery will eventually experience a recurrence of depressive symptoms (Mueller *et al*., 1999; Solomon *et al*., 2000). The risk of relapsing up to a year after remission is halved for patients who continue antidepressant treatment (ADT) compared to those who discontinue ADT (Sim *et al*., 2016). Treatment guidelines therefore recommend continuation of successful MDD treatment for at least 6 months after remission, with longer courses of treatment often recommended for patients with a history of recurrence (Bauer *et al*., 2013; Bauer *et al*., 2015).

Approximately 50% of patients with MDD have an inadequate response to first-line ADT (Rush *et al*., 2006). Adjunctive therapies are one option for patients who do not obtain adequate benefit from antidepressant monotherapy, and, among several proven adjunctive strategies, the evidence is most extensive for atypical antipsychotics (Connolly & Thase, 2011; Kishimoto *et al*., 2023). However, the longer-term safety and efficacy of adjunctive atypical antipsychotics in patients with MDD is not well established, and the few completed longer-term randomised controlled studies have yielded conflicting efficacy results (Rapaport *et al*., 2006; Brunner *et al*., 2014; Mohamed *et al*., 2017; Bauer *et al*., 2019).

Brexpiprazole is one of four atypical antipsychotics approved in the US for use as an adjunctive therapy to ADT for the treatment of MDD in adults (Riesenberg *et al*., 2023). The efficacy, safety, and tolerability of adjunctive brexpiprazole for the short-term treatment of MDD have been demonstrated in four 6-week Phase 3 randomised controlled trials (Thase *et al*., 2015a; Thase *et al*., 2015b; Hobart *et al*., 2018a; Hobart *et al*., 2018b). A longer-term randomised controlled trial found no meaningful difference between adjunctive brexpiprazole and adjunctive placebo on rate of remission over 24 weeks; these results may have been impacted by study design elements, such as a previously untested primary outcome, and the lack of a group switched from brexpiprazole to placebo after an acute response to adjunctive therapy, among other factors (Bauer *et al*., 2019).

The aim of the present withdrawal study was to compare time to relapse in patients stabilised on ADT + brexpiprazole who were then randomised to either continued adjunctive brexpiprazole or brexpiprazole withdrawal (a switch to adjunctive placebo). The safety and tolerability of adjunctive brexpiprazole for the maintenance treatment of MDD were also assessed.

## Methods

### Participants and study design

This was a Phase 3, multicentre, double-blind, placebo-controlled, parallel-arm, 26-week, randomised withdrawal study in patients with MDD (ClinicalTrials.gov identifier: NCT03538691). Patients were enrolled by investigators at 67 clinical trial sites in the US (70.5% of enrolled patients), Poland (21.8%), and Germany (7.7%).

The study enrolled male and female outpatients aged 18–65 years with a diagnosis of recurrent MDD as defined by the Diagnostic and Statistical Manual of Mental Disorders, Fifth edition (DSM-5); a current major depressive episode of ≥8 weeks in duration; and a 17-item Hamilton Depression Rating Scale (HAM-D_17_) total score of ≥18 at screening and baseline visits (Hamilton, 1960; Hamilton, 1967). At screening, patients were required to have a history of inadequate response to 1–2 ADTs during the current episode, plus a current inadequate response to an adequate duration of protocol-specified selective serotonin reuptake inhibitor (SSRI) or serotonin–noradrenaline reuptake inhibitor (SNRI) treatment, where inadequate response was defined as <50% improved as assessed by the Massachusetts General Hospital Antidepressant Treatment Response Questionnaire (Chandler *et al*., 2010). If required, the screening period could be used to achieve an adequate duration of SSRI/SNRI treatment. Thus, in total, eligible patients had an inadequate response to 2–3 ADTs during the current episode. Permitted SSRIs were citalopram hydrobromide tablets (20 or 40 mg/day), escitalopram tablets (10 or 20 mg/day), fluoxetine capsules (20 or 40 mg/day), paroxetine controlled-release tablets (37.5 or 50 mg/day), and sertraline tablets (100, 150, or 200 mg/day); permitted SNRIs were duloxetine delayed-release capsules (40 or 60 mg/day) and venlafaxine extended-release capsules (75, 150, or 225 mg/day).

Patients were excluded if they had another primary DSM-5 diagnosis (specified disorders included schizophrenia and bipolar disorder); current DSM-5 personality disorder; suicidal ideation in the past 6 months or suicidal behaviour in the past 2 years; any clinically significant neurological or unstable medical condition; previously received adjunctive antipsychotic medication for ≥3 weeks during the current episode; previous exposure to brexpiprazole; or received new-onset psychotherapy within 42 days of screening or during the trial.

**Figure 1.**
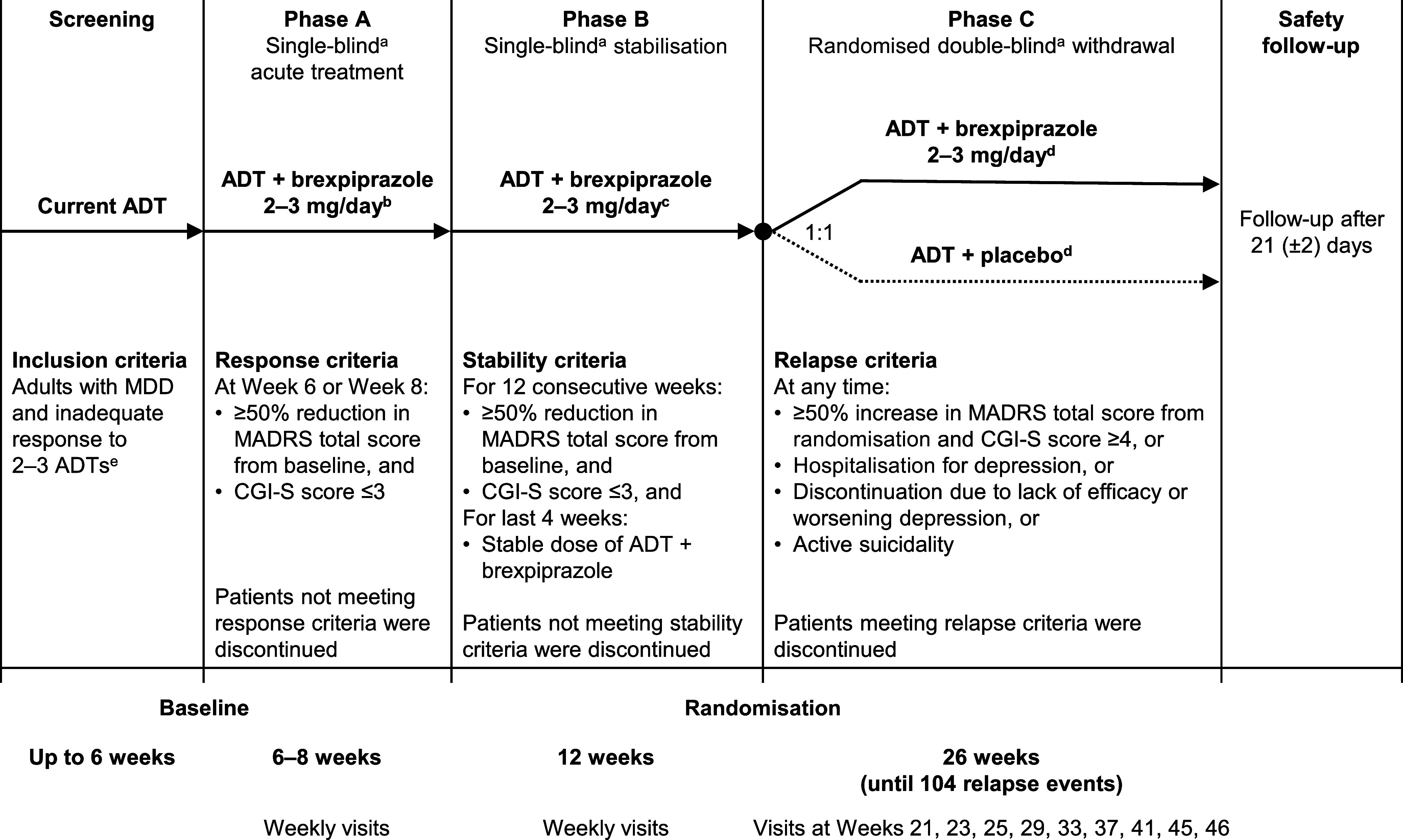
Study design. ADT, antidepressant treatment; CGI-S, Clinical Global Impression – Severity of illness; MADRS, Montgomery–Åsberg Depression Rating Scale; MDD, major depressive disorder. ^a^Blind applies to adjunctive treatment; patients continued on their current ADT, which was open-label throughout the study. ^b^Titrated as follows: first week, 0.5 mg/day; second week, 1 mg/day; third week, 2 mg/day; fourth week onwards, 2–3 mg/day (flexible dose). ^c^Flexible dose. ^d^One dose adjustment permitted. ^e^Specifically, history of inadequate response to 1–2 ADTs during the current episode, plus inadequate response to current ADT.

The trial consisted of a screening period, a 6–8-week acute treatment phase to identify patients who responded to ADT + brexpiprazole (Phase A), a 12-week stabilisation phase to stabilise patients on ADT + brexpiprazole (Phase B), a 26-week randomised double-blind parallel-group withdrawal phase (Phase C), and a safety follow-up (Fig. 1).

In Phase A, eligible patients continued their current ADT from screening (open-label), and brexpiprazole 2–3 mg/day was initiated (blinded to patients; dose titrated over 2–4 weeks). At Weeks 6 and 8, patients were evaluated for response using the following criteria (blinded to investigators): ≥50% reduction in Montgomery–Åsberg Depression Rating Scale (MADRS) total score from baseline (Montgomery & Åsberg, 1979), and Clinical Global Impression – Severity of illness (CGI-S) score of ≤3 (Guy, 1976). Patients who met the response criteria at either visit immediately entered Phase B, whereas patients who did not meet the criteria by the Week 8 visit were discontinued from the trial.

In Phase B, eligible patients continued ADT (open-label) + brexpiprazole 2–3 mg/day (blinded to patients). Stability was assessed using the response criteria from Phase A (blinded to investigators); patients were required to meet these criteria for 12 consecutive weeks (three excursions were permitted, provided they were non-consecutive and not at the last visit of Phase B). In addition, the dose of ADT + brexpiprazole had to be stable for the last 4 weeks of Phase B. Patients who met the stability criteria entered Phase C, whereas patients who did not meet the criteria were discontinued from the trial.

In Phase C, eligible patients continued ADT (open-label) and were randomised (1:1) to continued adjunctive brexpiprazole 2–3 mg/day or a switch to adjunctive placebo. Randomised treatments were assigned using a computer-generated randomisation code provided by the sponsor and stratified by site (block size 4). Treatment assignments were blinded to patients, investigators, and sponsor personnel, including those involved in data analysis. The transition from Phase B to Phase C was not made evident to patients. Study drugs were taken orally once daily at the same time each day, without regard to meals. Brexpiprazole tablets and matching placebo tablets were provided by the sponsor, packaged in blister cards labelled with patient and compound identification codes. Patients were assessed for relapse (defined in the ‘Endpoints’ section) at 2-weekly visits for the first two visits and at 4-weekly visits thereafter. Patients who met criteria for relapse were immediately discontinued from the trial and received alternative depression treatment.

### Endpoints

The primary endpoint was time to relapse in Phase C, defined as meeting any of the following criteria, measured from randomisation and blinded to investigators: (1) at the same visit, ≥50% increase in MADRS total score from randomisation and CGI-S score ≥4; (2) hospitalisation for depression; (3) discontinuation due to lack of efficacy or worsening of depression; (4) active suicidality, defined as a score of ≥4 on MADRS item 10, an answer of “yes” on question 4 or 5 on the Columbia Suicide Severity Rating Scale (C-SSRS), or an answer of “yes” to any of the questions on the Suicidal Behavior section of the C-SSRS (Posner *et al*., 2011).

Secondary endpoints were time to functional relapse in Phase C, proportion of patients meeting relapse criteria in Phase C, proportion of patients meeting remission criteria in Phase C, change in MADRS total score (which ranges from 0 [no depression] to 60 [very severe depression]) (Montgomery & Åsberg, 1979), change in CGI-S score (which ranges from 1 [normal, not at all ill] to 7 [among the most extremely ill patients]) (Guy, 1976), and change in Sheehan Disability Scale (SDS) Mean and item scores (Sheehan & Sheehan, 2008). The SDS is a measure of functional disability across three items – work/school (which is skipped if the patient is not currently attending work/school), social life, and family life – each scored from 0 (not at all disrupted) to 10 (extremely disrupted) (Sheehan & Sheehan, 2008), where SDS Mean score is the mean of the two or three completed items, and SDS total score is the sum of the two or three completed items. Functional relapse was defined as meeting all the following criteria at a single visit: ≥30% increase in SDS Mean score from randomisation, at least one SDS item with a score ≥4, and SDS total score ≥7 (if 3 items scored) or ≥5 (if the work/school item was not scored). Remission was defined as MADRS total score ≤10.

Safety was assessed via treatment-emergent adverse events (TEAEs), body weight, laboratory tests, vital signs, electrocardiograms (ECGs), the C-SSRS (Posner *et al*., 2011), and three extrapyramidal symptom rating scales: Simpson–Angus Scale (SAS) (Simpson & Angus, 1970), Abnormal Involuntary Movement Scale (AIMS) (Guy, 1976), and Barnes Akathisia Rating Scale (BARS) (Barnes, 1989). A TEAE was defined as an adverse event that started after initiation of trial medication, or an adverse event that continued from baseline of a specific phase and became serious, worsened, trial drug-related, or resulted in death, discontinuation, or interruption/reduction of trial medication during the same phase. TEAEs were coded using Medical Dictionary for Regulatory Activities (MedDRA) preferred terms.

### Statistical analysis

Time to relapse in Phase C was analysed in the efficacy sample, composed of all patients who took at least one dose of randomised study drug in Phase C. A two-sided log-rank test was used to test the differences in Kaplan–Meier survival curves. A 95% confidence interval (CI) for the hazard ratio (ADT + brexpiprazole vs. ADT + placebo) was calculated using a Cox proportional hazard model with treatment as the fixed effect. Patients who withdrew early from the trial without relapse or who were still in the trial at the end of Phase C (Week 46) were considered as censored observations at the discontinuation or completion date. Time to relapse was also analysed in the following subgroups: sex (male, female), race (White, all other races), age at Phase C baseline (i.e., at randomisation; <45 years, ≥45 years), and region (US, Europe).

Based on a prior randomised withdrawal trial of olanzapine/fluoxetine combination (Brunner *et al*., 2014), 104 relapse events were needed in the present trial to reach 95% power to test a hazard ratio of 0.49 at a two-sided alpha level of 0.05. This corresponded to a Phase C randomisation target of approximately 450 patients and a Phase A enrolment target of approximately 1450 patients. The randomisation and enrolment targets were approximate; the only hard target was 104 relapse events.

Two interim analyses were performed by an independent data monitoring committee after approximately 50% and 75% of relapse events had occurred. To keep the overall significance level at 0.05, the two-sided alpha levels for the two interim analyses were set at 0.0031 and 0.018, respectively, leaving 0.044 for the final analysis. The interim analyses did not meet the stopping criteria, and the committee recommended to continue the trial.

For comparisons of time to functional relapse (secondary endpoint), a log-rank test was used, similar to the primary analysis of time to relapse.

In Phases A and B, mean rating scale score changes from baseline were analysed using descriptive statistics (observed cases). Baseline was defined as the first visit of Phase A; Phase B did not have a separate baseline and the baseline for Phase A was used. For Phase C, the last value from Phase B was used as the baseline value, termed ‘randomisation’ in this article. In Phase C, mean rating scale score changes from randomisation were analysed via two approaches: (1) last observation carried forward (LOCF) using an analysis of covariance model; and (2) exploratory analysis using a mixed model for repeated measures (MMRM) with fixed effects of treatment, pooled centre, visit, treatment–visit interaction, baseline value, and baseline–visit interaction as covariate, with an unstructured variance–covariance matrix structure. MMRM analyses are considered superior to LOCF analyses of covariance for longitudinal studies in terms of minimising biases (Siddiqui *et al*., 2009). Secondary endpoints were tested at a nominal 0.05 level with no adjustment for multiplicity.

After the trial began, two efficacy endpoints (mean change in SDS Mean score from randomisation to Week 46, and time to functional relapse) were reclassified from ‘key secondary endpoints’ to ‘secondary endpoints’ in the final statistical analysis plan (May 13, 2021) following a discussion with the US Food and Drug Administration, which concluded that these endpoints might be influenced by the anticipated dropout associated with the primary endpoint.

A safety sample was defined for each phase, composed of all patients who took at least one dose of study drug during that phase. Safety results are presented using descriptive statistics.

All analyses were performed using SAS version 9.4 (SAS Institute Inc; Cary, NC).

## Results

### Patients

The trial began on July 13, 2018, and was terminated by the sponsor on June 15, 2022, upon reaching the target number of relapse events (106 relapse events were recorded; last follow-up date: July 13, 2022).

#### Phase A: single-blind acute treatment

**Figure 2.**
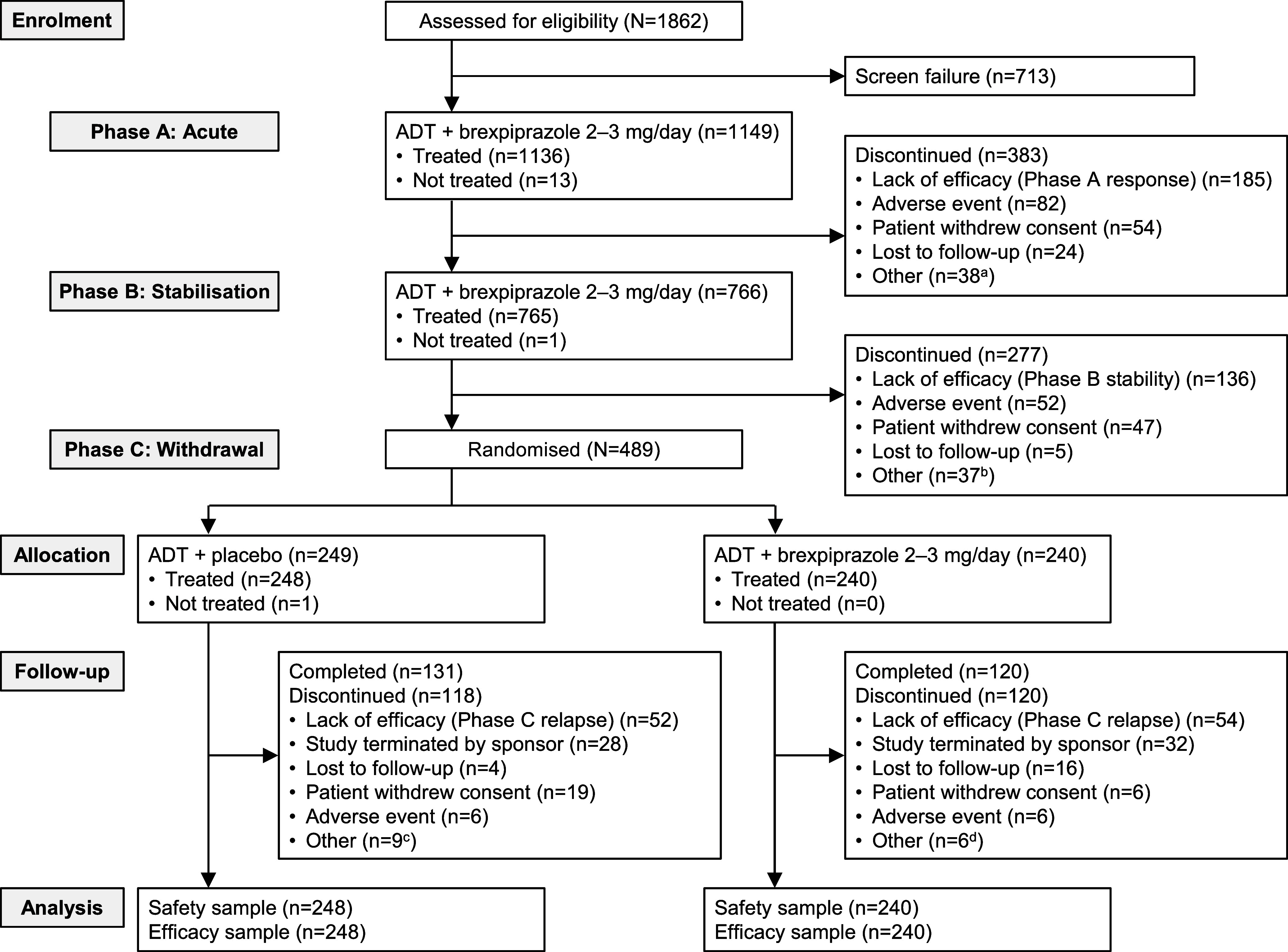
Patient disposition. ADT, antidepressant treatment. ^a^Protocol deviation (*n* = 12), non-compliance with study drug (*n* = 9), death (*n* = 1), physician decision (*n* = 1), other (*n* = 15). ^b^Non-compliance with study drug (*n* = 9), protocol deviation (*n* = 9), physician decision (*n* = 6), pregnancy (*n* = 1), COVID-19 restrictions (*n* = 1), other (*n* = 11). ^c^Non-compliance with study drug (*n* = 2), physician decision (*n* = 1), other (*n* = 6). ^d^Non-compliance with study drug (*n* = 1), pregnancy (*n* = 1), other (*n* = 4).

Overall, 1149 patients were enrolled in Phase A (Fig. 2), of whom 811 (70.6%) were taking an SSRI and 338 (29.4%) were taking an SNRI. Baseline demographic and clinical characteristics are presented in Table 1.

**Table 1.** Demographic and clinical characteristics at baseline (Phases A and B) and at randomisation (Phase C)

Characteristic^a^	Phase A: Acute treatment (enrolled sample)	Phase B: Stabilisation (enrolled sample)^b^	Phase C: Randomised withdrawal (randomised sample)	
	ADT + brexpiprazole (*n* = 1149)	ADT + brexpiprazole (*n* = 766)	ADT + brexpiprazole (*n* = 240)	ADT + placebo (*n* = 249)
**Demographics**				
Age, mean (SD), years	42.3 (13.1)	43.0 (13.3)	43.3 (13.0)	44.7 (13.7)
Weight, mean (SD), kg	85.0 (21.0)	86.0 (21.1)	88.0 (21.6) (*n* = 238)	88.4 (22.5) (*n* = 246)
BMI, mean (SD), kg/m^2^	29.7 (7.0)	30.0 (7.1)	30.6 (7.1) (*n* = 238)	30.9 (7.7) (*n* = 246)
Sex				
Female	734 (63.9)	495 (64.6)	162 (67.5)	159 (63.9)
Male	415 (36.1)	271 (35.4)	78 (32.5)	90 (36.1)
Race				
American Indian or Alaska Native	5 (0.4)	3 (0.4)	2 (0.8)	1 (0.4)
Asian	22 (1.9)	13 (1.7)	2 (0.8)	4 (1.6)
Black or African American	159 (13.8)	97 (12.7)	35 (14.6)	27 (10.8)
Native Hawaiian or other Pacific Islander	5 (0.4)	2 (0.3)	1 (0.4)	1 (0.4)
White	868 (75.5)	585 (76.4)	180 (75.0)	194 (77.9)
Other	90 (7.8)	66 (8.6)	20 (8.3)	22 (8.8)
Ethnicity				
Hispanic or Latino	110 (9.6)	71 (9.3)	21 (8.8)	19 (7.6)
Not Hispanic or Latino	968 (84.2)	640 (83.6)	202 (84.2)	211 (84.7)
Other	70 (6.1)	54 (7.0)	17 (7.1)	18 (7.2)
Unknown	1 (0.1)	1 (0.1)	0 (0.0)	1 (0.4)
**Clinical characteristics**				
Age at MDD diagnosis, mean (SD), years	29.2 (12.6)	29.2 (12.5)	29.4 (12.3)	30.7 (13.1)
MADRS total score, mean (SD)	31.4 (4.7)	31.4 (4.7)	5.0 (3.9)	4.4 (3.7)
CGI-S score, mean (SD)	4.7 (0.7)	4.7 (0.7)	1.6 (0.7)	1.5 (0.7)
SDS Mean score, mean (SD)	6.6 (1.7) (*n* = 1113)	6.5 (1.7) (*n* = 744)	1.6 (1.8) (*n* = 239)	1.3 (1.7) (*n* = 244)
SDS work/school score	6.4 (2.2) (*n* = 937)	6.3 (2.3) (*n* = 613)	1.6 (2.0) (*n* = 215)	1.2 (1.7) (*n* = 220)
SDS social life score	6.8 (2.1) (*n* = 1118)	6.7 (2.1) (*n* = 749)	1.5 (2.0) (*n* = 239)	1.3 (1.8) (*n* = 246)
SDS family life score	6.5 (2.1) (*n* = 1118)	6.4 (2.0) (*n* = 748)	1.5 (1.9) (*n* = 239)	1.3 (1.9) (*n* = 244)

ADT, antidepressant treatment; BMI, body mass index; CGI-S, Clinical Global Impression – Severity of illness; MADRS, Montgomery–Åsberg Depression Rating Scale; MDD, major depressive disorder; SD, standard deviation; SDS, Sheehan Disability Scale.

a Data are expressed as *n* (%) unless otherwise indicated.

b Phase B did not have a separate baseline (baseline for Phase A was used).

Response criteria were met at Weeks 6, 7 or 8 by 777/1149 enrolled patients (67.6%), of whom 766 (66.7%) continued into Phase B. The remaining 383 patients (33.3%) discontinued the study, most commonly (>5%) due to not meeting response criteria (16.1%), or adverse events (7.1%) (Fig. 2). Mean brexpiprazole dose at the end of Week 6 was 2.2 mg (*n* = 997).

#### Phase B: single-blind stabilisation

Stabilisation criteria were met at Week 20 by 489/766 patients (63.8%), all of whom continued into Phase C. The remaining 277 patients (36.2%) discontinued the study, most commonly (>5%) due to not meeting stabilisation criteria (17.8%), adverse events (6.8%), or withdrawal of consent (6.1%) (Fig. 2). Mean brexpiprazole dose at the end of Week 20 was 2.3 mg (*n* = 504).

#### Phase C: randomised double-blind withdrawal

In total, 489 patients were randomised in Phase C: 240 to ADT + brexpiprazole and 249 to ADT + placebo. Demographic and clinical characteristics at randomisation were similar between treatment groups (Table 1). The study was completed by 251/489 (51.3%) patients: 120/240 (50.0%) on ADT + brexpiprazole and 131/249 (52.6%) on ADT + placebo. The most common reasons for discontinuation (>5%) overall in Phase C were relapse (21.7%), the sponsor terminating the study upon reaching the target number of relapse events (12.3%), and withdrawal of consent (5.1%) (Fig. 2). Mean brexpiprazole dose at the end of Week 46 was 2.2 mg (*n* = 116).

### Efficacy

#### Phase A: single-blind acute treatment

**Figure 3.**
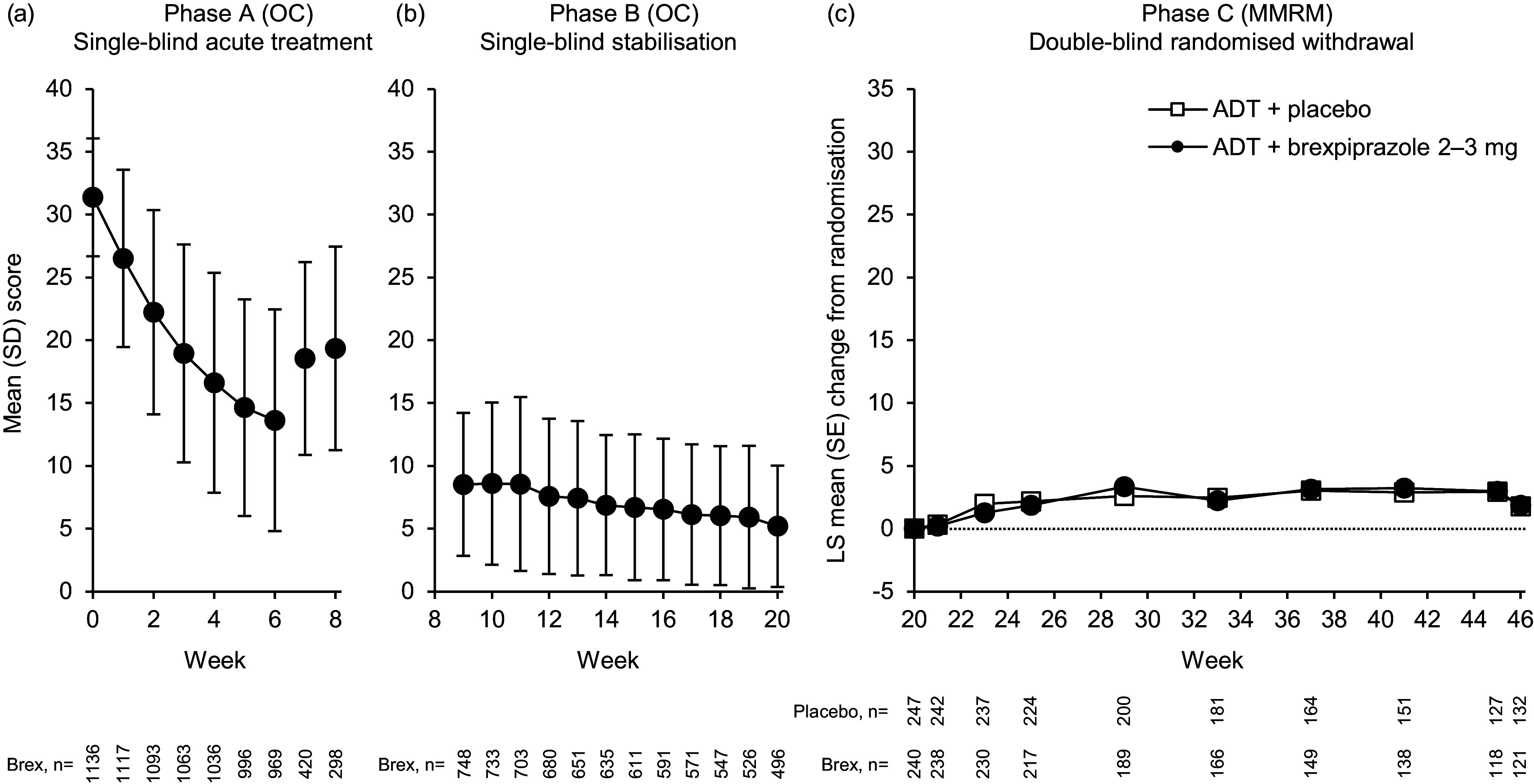
(***a***–***b***) Mean MADRS total score across Phases A and B. Values at Phase A Weeks 7 and 8 are for the subgroup of patients who did not meet response criteria at Week 6. (***c***) LS mean change from randomisation in mean MADRS total score in Phase C (efficacy sample). ADT, antidepressant treatment; MADRS, Montgomery–Åsberg Depression Rating Scale; LS, least squares; MMRM, mixed model for repeated measures; OC, observed cases; SD, standard deviation; SE, standard error.

Depression and functioning rating scale scores improved with ADT + brexpiprazole treatment during Phase A. The mean (standard deviation [SD]) change from Phase A baseline to Week 6 in MADRS total score was −17.7 (9.1) (*n* = 969) (Fig. 3a). The corresponding mean (SD) change in CGI-S score was −1.9 (1.2) (*n* = 969), and in SDS Mean score was −3.2 (2.5) (*n* = 882).

#### Phase B: single-blind stabilisation

Among the 766 patients who responded in Phase A and entered Phase B, depression and functioning rating scale scores continued to improve throughout the 12-week stabilisation phase with ADT + brexpiprazole. The mean (SD) change from Phase A baseline to Week 20 in MADRS total score was −26.1 (6.3) (*n* = 496) (Fig. 3b). The corresponding mean (SD) change in CGI-S score was −3.1 (1.0) (*n* = 497), and in SDS Mean score was −5.0 (2.3) (*n* = 460).

#### Phase C: randomised double-blind withdrawal

**Figure 4.**
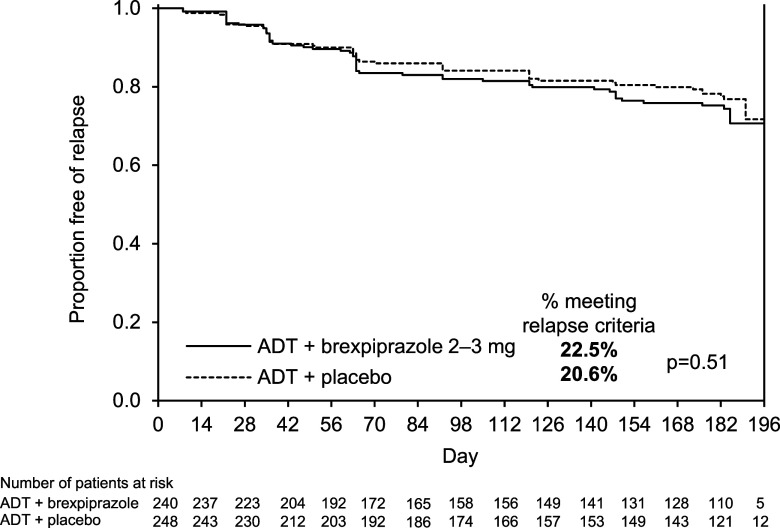
Kaplan–Meier product limit plot of time to relapse from randomisation in Phase C (primary efficacy endpoint, efficacy sample). ADT, antidepressant treatment.

One hundred and five relapse events were recorded in the efficacy sample; one additional relapse event occurred in a patient who was randomised but did not receive treatment in this phase. Time to relapse (primary endpoint) was similar between the ADT + brexpiprazole and ADT + placebo groups: median 63 days from randomisation in both groups (Fig. 4). The proportion of patients meeting relapse criteria during Phase C was also similar between treatment groups: 22.5% on ADT + brexpiprazole and 20.6% on ADT + placebo (*p* = 0.51) (Table 2). In the subgroup analyses by sex, race, age, and region, the 95% CIs of hazard ratios for time to relapse in each subgroup included 1, indicating no meaningful differences between ADT + brexpiprazole and ADT + placebo (data not shown).

**Table 2.** Summary of efficacy endpoints in Phase C (efficacy sample)

					Treatment difference	
Endpoint	Treatment group	*N*	*n* (%)		Hazard ratio (95% CI)^a^	*p*-value
Met relapse criteria	ADT + brexpiprazole	240	54 (22.5)		1.14 (0.78, 1.67)	0.51^b^
	ADT + placebo	248	51 (20.6)			
Met functional relapse criteria	ADT + brexpiprazole	239	81 (33.9)		1.18 (0.86, 1.62)	0.31^b^
	ADT + placebo	243	73 (30.0)			
Met remission criteria at last visit	ADT + brexpiprazole	240	173 (72.1)		–	0.85^c^
	ADT + placebo	247	180 (72.9)			
Endpoint	Treatment group	*N*	Randomisation (Week 20), mean (SD)	Change to Week 46, LS mean (SE)^d^	Treatment difference	
					LS mean difference (95% CI)^d^	*p*-value^d^
MADRS total	ADT + brexpiprazole	240	5.0 (3.9)	1.9 (0.5)	0.09 (-1.33, 1.51)	0.90
	ADT + placebo	247	4.4 (3.7)	1.8 (0.5)		
CGI-S	ADT + brexpiprazole	240	1.6 (0.7)	0.2 (0.1)	0.07 (-0.12, 0.26)	0.47
	ADT + placebo	247	1.5 (0.7)	0.2 (0.1)		
SDS Mean	ADT + brexpiprazole	239	1.6 (1.8)	0.2 (0.1)	0.31 (-0.03, 0.65)	0.070
	ADT + placebo	242	1.3 (1.7)	−0.1 (0.1)		
SDS work/school score	ADT + brexpiprazole	210	1.6 (2.0)	0.0 (0.2)	0.21 (-0.14, 0.57)	0.24
	ADT + placebo	210	1.2 (1.7)	−0.3 (0.1)		
SDS social life score	ADT + brexpiprazole	239	1.5 (2.0)	0.3 (0.2)	0.35 (-0.03, 0.73)	0.073
	ADT + placebo	244	1.3 (1.8)	0.0 (0.2)		
SDS family life score	ADT + brexpiprazole	239	1.5 (1.9)	0.2 (0.1)	0.28 (-0.06, 0.62)	0.10
	ADT + placebo	242	1.4 (1.9)	−0.1 (0.1)		

ADT, antidepressant treatment; CI, confidence interval; CGI-S, Clinical Global Impression – Severity of illness; LS, least squares; MADRS, Montgomery–Åsberg Depression Rating Scale; MMRM, mixed model for repeated measures; SD, standard deviation; SDS, Sheehan Disability Scale; SE, standard error.

a Cox proportional hazard model. Hazard ratio <1 is in favour of ADT + brexpiprazole.

b Log-rank test.

c Chi-square test.

d MMRM.

Time to functional relapse was similar between treatment groups: median 36 days for ADT + brexpiprazole and 35 days for ADT + placebo. The proportion of patients meeting functional relapse criteria during Phase C was also similar between treatment groups: 33.9% on ADT + brexpiprazole and 30.0% on ADT + placebo (*p* = 0.31) (Table 2). Remission criteria were met by a similar proportion of patients at their last visit in both treatment groups: 72.1% on ADT + brexpiprazole and 72.9% on ADT + placebo (*p* = 0.85) (Table 2).

From randomisation to Week 46, MADRS total score changes were small, with no significant differences between treatment groups (Table 2, Fig. 3c [MMRM]; Supplementary Table S1 [LOCF]). Similarly, there were no significant differences between treatment groups in change from randomisation to Week 46 in CGI-S score, SDS Mean score, and SDS item scores (Table 2 [MMRM]; Supplementary Table S1 [LOCF]).

### Safety

The incidences of TEAEs, serious TEAEs, and discontinuation due to TEAEs in each phase are presented in Table 3.

**Table 3.** Summary of treatment-emergent adverse events (safety sample)

Event^a^	Phase A: Acute treatment	Phase B: Stabilisation	Phase C: Randomised withdrawal	
	ADT + brexpiprazole (*n* = 1136)	ADT + brexpiprazole (*n* = 765)	ADT + brexpiprazole (*n* = 240)	ADT + placebo (*n* = 248)
At least one TEAE	751 (66.1)	431 (56.3)	102 (42.5)	103 (41.5)
At least one serious TEAE	10 (0.9)	12 (1.6)	1 (0.4)	3 (1.2)
Discontinuation due to TEAE	82 (7.2)^b^	51 (6.7)^c^	7 (2.9)^d^	6 (2.4)^d^
Death	1 (0.1)^e^	0 (0.0)	0 (0.0)	0 (0.0)
TEAEs with an incidence ≥5% in any treatment group in any phase				
Increased weight	48 (4.2)	122 (15.9)	25 (10.4)	13 (5.2)
Somnolence	90 (7.9)	37 (4.8)	6 (2.5)	3 (1.2)
Headache	113 (9.9)	41 (5.4)	5 (2.1)	14 (5.6)
Akathisia	109 (9.6)	33 (4.3)	4 (1.7)	3 (1.2)
Increased appetite	60 (5.3)	18 (2.4)	1 (0.4)	4 (1.6)
Insomnia	72 (6.3)	8 (1.0)	1 (0.4)	2 (0.8)
Nausea	64 (5.6)	18 (2.4)	1 (0.4)	0 (0.0)
Other TEAEs of interest				
Anxiety	34 (3.0)	19 (2.5)	6 (2.5)	1 (0.4)
Restlessness	54 (4.8)	13 (1.7)	2 (0.8)	1 (0.4)
Extrapyramidal disorder	1 (0.1)	2 (0.3)	1 (0.4)	0 (0.0)
Sedation	24 (2.1)	2 (0.3)	0 (0.0)	0 (0.0)

ADT, antidepressant treatment; TEAE, treatment-emergent adverse event.

a Data are expressed as n (%).

b Most commonly (>1%) akathisia, *n* = 21 (1.8%).

c Most commonly (>1%) akathisia, *n* = 11 (1.4%).

d No TEAE led to discontinuation in >1% of patients.

e Cardiac arrest.

#### Phase A: single-blind acute treatment

TEAEs with an incidence ≥5% during Phase A (6–8 weeks) were headache (9.9%), akathisia (9.6%), somnolence (7.9%), insomnia (6.3%), nausea (5.6%), and increased appetite (5.3%). Most TEAEs in Phase A were mild or moderate in severity; severe TEAEs were reported by 3.2% of patients.

One death was reported in Phase A; this was the only death in the study. The patient, a 27-year-old White male taking citalopram + brexpiprazole, died of cardiac arrest on Day 12. The patient had a history of illicit drug use, but no history of cardiac disorder, and ECG measurements at screening and baseline were normal. The patient became ill after returning from a vacation during which he had not taken citalopram + brexpiprazole for 3–4 days. The death was considered by the investigator to be unrelated to study drug.

The mean (SD) weight change from Phase A baseline to Phase A last visit (up to 8 weeks) was + 1.2 (2.3) kg. The incidence of potentially clinically relevant weight gain (≥7%) at any visit in Phase A was 4.3% (48/1121), and the corresponding incidence of potentially clinically relevant weight loss (≥7%) was 0.8% (9/1121). In general, mean changes in laboratory parameters (including metabolic parameters), vital signs, and ECG findings over time were small and not clinically meaningful in all three phases (data not shown). Of note, the mean (SD) change in fasting triglycerides from Phase A baseline to Phase A last visit (up to 8 weeks) was + 18.9 (148.0) mg/dL.

The incidence of suicidal ideation as a TEAE was 0.3% (3/1136) in Phase A, and there was no indication of treatment-emergent suicidal behaviour as measured by the C-SSRS. Extrapyramidal symptom rating scale scores (SAS, AIMS, and BARS) showed little change from Phase A baseline to last visit of Phase A (all <0.1 points) (data not shown).

#### Phase B: single-blind stabilisation

TEAEs with an incidence ≥5% during Phase B (12 weeks) were increased weight (15.9%) and headache (5.4%). Most TEAEs in Phase B were mild or moderate in severity; severe TEAEs were reported by 2.6% of patients.

The mean (SD) weight change from Phase A baseline to Phase B last visit (up to 20 weeks) was + 2.2 (4.0) kg. The incidence of potentially clinically relevant weight gain (≥7%) at any visit in Phase B was 23.2% (173/745), and the corresponding incidence of potentially clinically relevant weight loss (≥7%) was 2.4% (18/745). The mean (SD) change in fasting triglycerides from Phase A baseline to Phase B last visit (up to 20 weeks) was + 14.1 (78.8) mg/dL.

The incidence of suicidal ideation as a TEAE was 0.5% (4/765) in Phase B. There was one TEAE of suicide attempt during Phase B; this was the only suicide attempt in the study. The patient consumed 20 over-the-counter sleeping pills in the evening; the next morning, the patient woke up at the normal time and in retrospect was glad that the suicide attempt did not work. The patient continued in the trial with no dose changes. On the C-SSRS, suicidal behaviour was reported by two patients in Phase B (0.3%), and an actual attempt, aborted attempt, and preparatory acts or behaviour were each reported in one patient (0.1%). Extrapyramidal symptom rating scale scores (SAS, AIMS, and BARS) showed little change from Phase A baseline to last visit of Phase B (all <0.1 points) (data not shown).

#### Phase C: randomised double-blind withdrawal

The incidences of TEAEs, serious TEAEs, and discontinuation due to TEAEs were similar between treatment groups in Phase C (26 weeks) (Table 3). The only TEAE with an incidence ≥5% in the ADT + brexpiprazole group during Phase C was increased weight (10.4%, vs. 5.2% in the ADT + placebo group). In the ADT + placebo group, headache also had incidence ≥5% during Phase C (5.6%). Most TEAEs in Phase C were mild or moderate in severity; severe TEAEs were reported by 1.7% of patients in the ADT + brexpiprazole group and 2.0% in the ADT + placebo group.

In Phase C, the mean (SD) weight change from randomisation to last visit (up to 26 weeks) was 0.0 (3.4) kg in the ADT + brexpiprazole group and −1.0 (3.7) kg in the ADT + placebo group. The incidence of potentially clinically relevant weight gain (≥7%) at any visit in Phase C was 5.5% (13/235) with ADT + brexpiprazole and 2.9% (7/243) with ADT + placebo. The corresponding incidence of potentially clinically relevant weight loss (≥7%) was 5.1% (12/235) with ADT + brexpiprazole and 7.8% (19/243) with ADT + placebo. In contrast to Phases A and B, fasting triglycerides decreased during Phase C: the mean (SD) change from randomisation to last visit (up to 26 weeks) was −5.9 (68.3) mg/dL in the ADT + brexpiprazole group and −15.8 (80.3) mg/dL in the ADT + placebo group.

The incidence of suicidal ideation as a TEAE in Phase C was 0% (0/240) with ADT + brexpiprazole and 0.4% (1/248) with ADT + placebo, and there was no indication of treatment-emergent suicidal behaviour as measured by the C-SSRS. Extrapyramidal symptom rating scale scores (SAS, AIMS, and BARS) showed little change from randomisation to last visit of Phase C (all <0.1 points) (data not shown).

## Discussion

In this randomised withdrawal study, continued adjunctive brexpiprazole treatment did not differ from brexpiprazole withdrawal (i.e., switch to adjunctive placebo) on the primary endpoint of time to relapse. This may be attributed to the observed high rate of sustained response in both randomised groups. Regardless of whether patients continued adjunctive brexpiprazole or switched to adjunctive placebo in the randomised withdrawal phase (Phase C), approximately 80% of patients who were stabilised with ADT + brexpiprazole remained relapse free at their last visit (up to 26 weeks), and approximately 70% met remission criteria at their last visit.

Although no additional benefit of adjunctive brexpiprazole maintenance treatment was demonstrated versus brexpiprazole withdrawal, important improvements were observed in acutely ill patients treated with adjunctive brexpiprazole. Specifically, high rates of response (approximately two thirds of patients responded in Phase A) and stabilisation (approximately two thirds of patients who responded in Phase A became stable in Phase B) were observed, together with large mean improvements on depressive symptom and functioning rating scale scores, in line with previous short-term trials of ADT + brexpiprazole (Thase *et al*., 2015a; Thase *et al*., 2015b; Hobart *et al*., 2018a; Hobart *et al*., 2018b; Thase *et al*., 2019; Hobart *et al*., 2019a). In Phase A, a mean 17.7-point improvement in MADRS total score was observed over 6 weeks – a meaningful improvement, especially in patients who have previously proven difficult to treat (Hudgens *et al*., 2021; Turkoz *et al*., 2021). Overall, Phase A and B data suggest that adjunctive brexpiprazole treatment is beneficial over up to 20 weeks in the majority of patients with inadequate response to ADT. Furthermore, rating scale changes were small in Phase C, indicating that early improvement of depressive symptoms and functioning was maintained in the majority of stabilised patients.

Two other adjunctive antipsychotics have been investigated in randomised withdrawal studies in patients with ‘treatment-resistant’ MDD, for which definitions vary (Rapaport *et al*., 2006; Brunner *et al*., 2014; McIntyre *et al*., 2023). In the first study, adjunctive risperidone did not significantly differ from risperidone withdrawal (adjunctive placebo) on time to relapse (Rapaport *et al*., 2006). In the second study, olanzapine/fluoxetine combination demonstrated significantly longer time to relapse than olanzapine withdrawal (fluoxetine monotherapy) (Brunner *et al*., 2014). Although cross-trial comparisons must be made with caution due to differences in trial design, conduct, and population, it is notable that the relapse rate in the antipsychotic withdrawal arm was higher in the olanzapine/fluoxetine combination study (31.8% over 27 weeks) than in the present study (20.6% over 26 weeks). There are two sources of bias in the olanzapine/fluoxetine combination study that may have contributed to the increased relapse rate in the antipsychotic withdrawal arm. First, it is possible that the disappearance of characteristic side effects of olanzapine, notably sedation/somnolence (Eugene *et al*., 2021), in the antipsychotic withdrawal arm may have resulted in awareness of treatment allocation and functional unblinding. Second, many antidepressants and antipsychotics are associated with withdrawal symptoms such as agitation and anxiety when discontinuing treatment, which may be considered a marker of relapse (Fava *et al*., 2015; Fava *et al*., 2018; Brandt *et al*., 2022). Brexpiprazole has a longer steady-state half-life than olanzapine (91 vs. 30 hours) (Rexulti (brexpiprazole) Prescribing Information, 2023; Symbyax (olanzapine and fluoxetine) Prescribing Information, 2023; Callaghan *et al*., 1999), which may suggest that brexpiprazole is less likely to be associated with withdrawal symptoms than olanzapine (Andrade, 2022). Indeed, the only TEAE with incidence ≥5% in the brexpiprazole withdrawal group (and not in the continued brexpiprazole group) was headache, with incidence 5.6%.

Overall, safety data were consistent with earlier studies of longer-term brexpiprazole therapy (Nelson *et al*., 2016; Weiss *et al*., 2018; Bauer *et al*., 2019; Newcomer *et al*., 2019; Hobart *et al*., 2019b) and indicate that longer-term treatment with adjunctive brexpiprazole is generally well tolerated. The most frequent TEAEs during acute treatment (headache, akathisia, somnolence, insomnia, nausea, and increased appetite) all had a reduced frequency in Phases B and C, indicating either that these events were transient, or were less common among patients who responded to acute treatment. The exception was weight gain, which was potentially clinically relevant in 23.2% of patients in Phase B, and which increased by a mean of 2.2 kg from baseline to end of Phase B, before stabilising in Phase C. In the olanzapine/fluoxetine combination study, 34.8% of patients had potentially clinically relevant weight gain prior to randomisation, and mean weight gain from baseline to the end of Phase B was 4.2 kg (Brunner *et al*., 2014).

One patient died during the present study (0.1%) – a comparable rate to that in prior longer-term studies of adjunctive brexpiprazole in MDD (Bauer *et al*., 2019; Hobart *et al*., 2019b). In general, the risk of suicidal behaviour is low in controlled studies of treatments for depression (Stone *et al*., 2009) and, consistent with this, there was one suicide attempt (0.1%) and two cases of suicidal behaviour (0.3%) in the present study. Rates of suicidal behaviour were also low (<1%) in the prior longer-term studies of adjunctive brexpiprazole (Bauer *et al*., 2019; Hobart *et al*., 2019b).

Considering study limitations, participants received 18–20 weeks of adjunctive brexpiprazole therapy before randomisation; risk of relapse may have been higher if a shorter duration of adjunctive therapy was provided prior to randomisation. This study is also limited because there was no comparator arm in Phases A and B, meaning that the large benefits observed in these phases cannot be attributed to adjunctive brexpiprazole with certainty. In Phase C, there was no active comparator arm (such as another adjunctive antipsychotic) to validate the design and conduct of the trial. Relapse rates were low in the group randomised to brexpiprazole withdrawal, which may suggest that the study lacked sensitivity. Secondary outcomes in Phase C were not adjusted for multiplicity. The results of secondary outcomes might have been influenced by the dropout associated with the primary endpoint; for example, patients who experienced symptomatic relapse prior to functional relapse may be censored in the functional relapse analysis, leading to an underestimate of the time to functional relapse. The study took place during the COVID-19 pandemic, meaning that some visits were virtual and therefore without scheduled vital sign assessments; however, this was not thought to impact the efficacy results. Finally, as with all clinical trials, inclusion criteria limit the generalisability to a broader patient population.

In conclusion, while this study found no difference in time to relapse with continued adjunctive brexpiprazole versus brexpiprazole withdrawal, data from Phases A and B showed that depressive symptoms and functioning improved in patients with MDD and inadequate response to ADT who received adjunctive brexpiprazole, and data from Phase C showed that approximately 80% of stabilised patients remained relapse free at their last visit. MDD is a heterogeneous disease, and treatment approaches and decisions regarding treatment discontinuation should be individualised for each patient. Safety results were consistent with prior adjunctive brexpiprazole studies, supporting that adjunctive brexpiprazole therapy is generally well tolerated over up to 46 weeks. Furthermore, after 18–20 weeks of treatment, discontinuation of adjunctive brexpiprazole in stabilised patients with MDD was associated with minimal adverse withdrawal effects. This may be reassuring to clinicians given that this treatment duration is in line with normal clinical practice in the US, where the average treatment duration with an adjunctive antipsychotic is 14–17 weeks (Gerhard *et al*., 2018).

## Supporting information

McIntyre et al. supplementary material McIntyre et al. supplementary material

## Supplementary material

The supplementary material for this article can be found at https://doi.org/10.1017/neu.2024.32.
