# Supplementary material for: A double-blind, placebo-controlled, randomised withdrawal study of adjunctive brexpiprazole maintenance treatment for major depressive disorder
Source: Acta Neuropsychiatr. 2024 Oct 17;37:e33. doi: 10.1017/neu.2024.32 (PMC13130322; doi:10.1017/neu.2024.32)
Supplement: McIntyre et al. supplementary material [file S0924270824000322sup001.pdf]

Supplementary material

Supplement to: A double-blind, placebo-controlled, randomized withdrawal study of adjunctive brexpiprazole maintenance treatment for major depressive disorder

Roger S. McIntyre, Kripa Sundararajan, Saloni Behl, Nanco Hefting, Na Jin, Claudette Brewer, Mary Hobart, Michael E. Thase

Table S1. Summary of efficacy rating scale endpoints in Phase C (efficacy sample), using the LOCF approach

Endpoint	Treatment group	N	Randomization (Week 20), mean (SD)	Change to Week 46, LS mean (SE)	Treatment difference	
					LS mean difference (95% CI)	p-value
MADRS total	ADT + brexpiprazole	240	5.0 (3.9)	4.1 (0.8)	-0.12 (-1.73, 1.49)	0.88
	ADT + placebo	247	4.4 (3.7)	4.2 (0.7)		
CGI-S	ADT + brexpiprazole	240	1.6 (0.7)	0.6 (0.1)	0.03 (-0.18, 0.24)	0.80
	ADT + placebo	247	1.5 (0.7)	0.5 (0.1)		
SDS Mean	ADT + brexpiprazole	239	1.6 (1.8)	0.7 (0.2)	0.23 (-0.16, 0.62)	0.24
	ADT + placebo	242	1.3 (1.7)	0.5 (0.2)		
SDS work/school score	ADT + brexpiprazole	210	1.6 (2.0)	0.5 (0.2)	0.15 (-0.31, 0.61)	0.52
	ADT + placebo	210	1.2 (1.7)	0.3 (0.2)		
SDS social life score	ADT + brexpiprazole	239	1.5 (2.0)	0.9 (0.2)	0.36 (-0.06, 0.77)	0.090
	ADT + placebo	244	1.3 (1.8)	0.6 (0.2)		
SDS family life score	ADT + brexpiprazole	239	1.5 (1.9)	0.8 (0.2)	0.25 (-0.16, 0.67)	0.23
	ADT + placebo	242	1.4 (1.9)	0.5 (0.2)		

ADT, antidepressant treatment; CGI-S, Clinical Global Impression – Severity of illness; CI, confidence interval; LOCF, last observation carried forward; LS, least squares; MADRS, Montgomery–Åsberg Depression Rating Scale; SD, standard deviation; SDS, Sheehan Disability Scale; SE, standard error.
